# Social participation reduces isolation among Japanese older people in urban area: A 3-year longitudinal study

**DOI:** 10.1371/journal.pone.0222887

**Published:** 2019-09-20

**Authors:** Manami Ejiri, Hisashi Kawai, Yoshinori Fujiwara, Kazushige Ihara, Yutaka Watanabe, Hirohiko Hirano, Hun Kyung Kim, Kaori Ishii, Koichiro Oka, Shuichi Obuchi

**Affiliations:** 1 Tokyo Metropolitan Institute of Gerontology, Tokyo, Japan; 2 Graduate School of Sport Sciences, Waseda University, Saitama, Japan; 3 Faculty of Medicine, Hirosaki University, Aomori, Japan; 4 Faculty of Sport Sciences, Waseda University, Saitama, Japan; University of Malaya, MALAYSIA

## Abstract

**Objectives:**

Social isolation is a particular problem among older people and social participation may reduce future isolation. However, it is unclear which types of activities and which level of participation are effective. This study examines the relationship between social participation and isolation among Japanese older people by employing a 3-year longitudinal study.

**Methods:**

A mail survey was sent to 3,518 community-dwelling older people in an urban area in 2014 (baseline: BL). We then conducted follow-up mail survey on respondents who were non-isolated at BL in 2017 (follow-up: FL), with isolation being defined as being in contact with others less than once a week. An analysis was carried out on 1,070 subjects (398 men and 672 women). Social participation is defined by participation in group activities (community, senior club, hobbies, sports, volunteering, politics, industry, and religion). A logistic regression analysis was conducted to determine the association between the types of social participation and the number of organization types at BL, and isolation at FL.

**Results:**

At FL, 75 men (18.8%) and 59 women (8.8%) were considered to be isolated. Among the men, participation in a hobby group and sports group both significantly reduced the degree of isolation. Moreover, participation in two organizations and three or more organizations significantly lowered the risk of isolation when compared to non-participants. Among women, there were no significant associations among particular types of social activities and isolation. On the other hand, participation in one organization and three or more organizations significantly reduced their isolation when compared to non-participants. There was a significant linear trend between the number of types of organizations and isolation, regardless of gender.

**Conclusions:**

Participation in social activities reduces future isolation in older people. Encouraging participation in social activities could help reduce negative health outcomes associated with social isolation later in life.

## Introduction

There is currently a growing interest in social issues globally, such as the isolation—as well as physical or mental health decline—of older people. Social isolation can be defined as an objective state where an individual has few close relationships, social ties, or contact with others in a community [1]. Due to changes within individuals’ social networks or reduced social roles associated with advancing age, social isolation is a particular problem among older people [2]. Socially isolated older people are at an increased risk of needing care [3, 4], of dementia [5, 6], of depression and sleep disturbances [7], low health-related quality of life [8] and mortality [8–14]. Previously, most studies have focused on social isolation as an independent variable that leads to negative health outcomes [2]. However, few studies have focused on social isolation itself as an outcome [2]. Therefore, we have to point out that knowledge concerning the predictive factors of isolation—which is necessary to develop preventive intervention—is limited [15]. Previous cross-sectional studies have reported that factors related to isolation later in life are; gender [16–18], living alone [1, 16, 19, 20], low income [18], and low subject or compromised mental health [16, 17, 21]. Additionally, one of the few longitudinal studies on this subject revealed that the low frequency of group participation in social activities is a predictor of isolation [22]. Therefore, encouragement of social participation may reduce old-age isolation.

Previous studies have also suggested that social participation may lower the risk of various negative health outcomes, such as all-cause mortality [23, 24], cognitive decline [25, 26] and depression [27, 28]. According to Levasseur et al. (2010), social participation can be defined as a person’s involvement in activities that provide interaction with others in society or in the community [29]. When considering the effect of social participation, it is necessary to focus on the type and number of activities. Concerning activity types, participation in hobbies or sports groups were more effective at decreasing the risk of disability than local community activities [30, 31]. These studies also suggested that the relationship between social participation and health outcomes varies according to the type of activity. Therefore, it is important to clarify what kind of activity participation is effective for maintaining health. Despite the extensive literature mentioned above, no study has been conducted to examine the type of activity and its effect on isolation. Concerning the amount of activity, previous studies have shown that participation in multiple organizations is effective in maintaining mental health or in preventing disability [28, 30]. However, the relationship between the amount of activity and its protective effect on isolation has not been clarified.

A number of previous studies revealed that there is a difference between the genders concerning the impact of social participation on geriatric health [23, 31–35]. Similarly, there is a difference concerning the gender of individuals and the risk of isolation, indicating that men are more likely to become isolated than women [16–18, 22]. However, no study has investigated the relationship between social activities and isolation while considering gender differences.

It is essential to identify which activity types and which levels of participation reduces future isolation. This information will enable the promotion of social participation among older people, leading in turn to the prevention of isolation. The purpose of this study is therefore to investigate the relationship between social participation and isolation while considering the different genders from the perspective of type of activity and number of activity organizations in a 3-year longitudinal study. To the best of our knowledge, no longitudinal study has been done that analyzes the effect of social participation on the isolation of older people. The results of this study will be beneficial in creating effective strategies to reduce isolation. To address the research question, we conducted a mail survey on Japanese older people living in an urban area that has high proportion of isolated older people compared to rural areas.

## Method

### Participants and procedure

**Fig 1:**
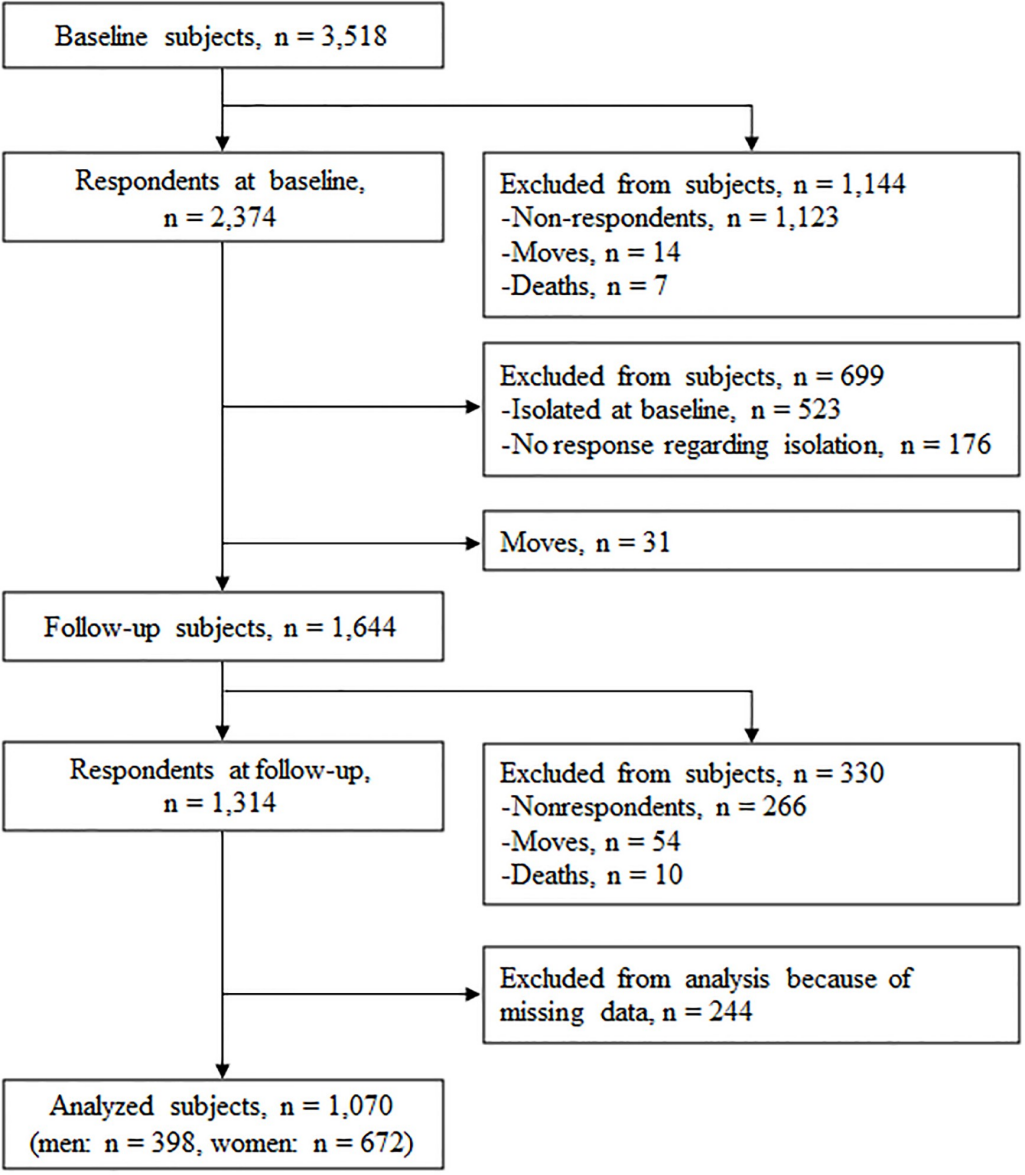
Flow diagram of study participant enrollment.

The present study is based on a cohort study of community-dwelling older people living in nine districts of Itabashi Ward in the urban area of Tokyo, Japan. The participants of the cohort study are 3,696 respondents of a mail survey for all residents aged from 65 to 85 living in the target area (n = 7,015) in 2012, and we follow up with these respondents virtually every year. In the present study, we distributed a self-administered questionnaire with questions concerning lifestyle and health status to 3,518 subjects in 2014, of which 2,374 replied (baseline: BL, response rate: 67.5%). Of these respondents, 523 subjects (22.0%) were already isolated at BL. In 2017, we followed up with 1,644 respondents who were non-isolated at BL by sending a questionnaire again and 1,314 replied (follow-up: FL). The final eligible study sample consisted of 1,070 respondents who had no missing values of measures (Fig 1). There were no significant differences with regards to age and gender between analyzed subjects and excluded subjects with missing data.

Ethical approval for the study was granted by the ethics committee of the Tokyo Metropolitan Institute of Gerontology (Acceptance no. 61, 2013). The purpose of the study and information privacy statement was provided in a briefing document along with the questionnaire and informed consent was requested from subjects, which they could provide when returning the questionnaire.

### Measurements

#### Dependent variable: Social isolation

Social isolation was defined based on the frequency of face-to-face contact and non-face-to-face contact (talking on the phone or communication via e-mail or letter) with non-resident family and friends. Respondents who answered that they had contact with both relatives and friends “less than once a week” were considered to be socially isolated [4].

#### Independent variable: Social participation

Social participation was defined as a person’s involvement in activities that provide interaction with others in their society or community [29]. For this study, we included questions concerning participation in the following eight group activities; neighborhood community associations (community), senior citizen clubs (senior club), hobby groups (hobby), sports groups (sports), volunteer groups (volunteer), political organizations or groups (politics), industrial or trade associations (industry), and religious organizations or groups (religion). The subjects could respond concerning whether or not they participated in each type of organization. The total number of organization types was calculated by adding up the number of organizations and classifying them into 4 categories: 0 (no participation), 1, 2 or > = 3, depending on its deviation [30]. Neighborhood community associations are organizations that are aimed at make creating a comfortable and safe environment within a community. Their activities include fire prevention activities, traffic safety, and community beautification [36]. According to a survey conducted by the Cabinet Office of the Government of Japan in 2017, approximately 20% of older people participate in neighborhood community associations in Japan [37]. However, this participation rate has decreased as city sizes increased [37]. Japanese senior clubs are established based on the Law on Social Welfare for the Elderly, legislated in 1963, and local governments provide assistance to these clubs. Their activities are mainly aimed at enriching the lives of older people through fun activities, such as studying, health promotions, recreation, and cultural activities. Senior clubs are considered to be a community-based bonding organization, similar to neighborhood community associations [38].

#### Covariates

Based on previous studies, the age, self-reported health, chronic conditions, instrumental activities of daily living disability (IADL disability), frequency of going outdoors, family structure, perceived financial status, and social support were used as covariates that may relate to social isolation. Self-reported health was categorized as good or poor. Chronic conditions were assessed using a checklist of eight diseases and conditions common in older individuals (high blood pressure, diabetes, stroke, cancer, liver disease, heart disease, dental problems, and orthopedic disorders). The IADL was evaluated by five questions based on the Tokyo Metropolitan Institute of Gerontology Index of Competence (TMIG-IC) [39]. The total number of answers concerning what the respondents are unable to do was used as the IADL disability value (score range from zero to five). Frequency of going outdoors was divided into two categories based on the definition of homeboundness; twice a week or more and once a week or less [40]. Family structure was categorized as either living alone or not [21]. The respondents’ perceived financial status was assessed as “hard” or not [22]. Emotional and instrumental social support were assessed by the total number of following options who would give support to the respondents: spouse, co-resident children, non-resident children and relatives, neighbors, friends, and others [41].

### Statistical analysis

The characteristics of the respondents that were deemed to be isolated at FL were compared using chi-square test and non-paired t-test. A logistic regression analysis was used to estimate the odds ratios (ORs) of isolation and 95% confidence intervals (CIs) associated with type of social participation and the number of organization types respectively. We fitted the following three models: Model 1 was a crude model that examined the independent association between each of the independent variables with no other variables. Model 2 was adjusted for age. Additionally, participation in all types of organizations was added only for “type of social participation.” Model 3 was adjusted for the covariates in Model 2 plus all the other covariates. In Model 3, a trend analysis was performed by the analysis of “number of organization types.” Prior to the logistic regression analysis, we did a correlation analysis among the independent variables to avoid multicollinearity. The coefficient of correlation was up to 0.33 (p < 0.001) between community and senior club. We therefore included all the independent variables in Model 2 and Model 3. In each model, non-participation was used as the reference category. All the analyses were performed stratified by gender. All the statistical analyses were carried out using SPSS statistics, version 23 (IBM, Chicago, IL, USA). The significance level was set at p < 0.05.

## Results

Of the 1,070 subjects (398 men and 672 women) that were analyzed, 75 men (18.8%) and 59 women (8.8%) were deemed to be isolated at FL. Table 1 shows the baseline characteristics of both genders by isolation states at FL. Isolated men were significantly older, participated less in community, hobby, sports, and politics groups, and participated in fewer organization types at BL than the non-isolated men (Table 1). The women deemed to be isolated at FL participated less in community, hobby, and volunteer groups and participated in fewer organization types at BL than non-isolated women (Table 1). Particularly, nearly half of both the isolated men and women groups participated in no group activities at BL, whereas the non-participation rate was between 20–30% among the non-isolated men and women.

**Table 1:** Baseline characteristics.

	Men (n = 398)					Women (n = 672)				
	non-isolated (n = 323)		isolated (n = 75)		p	non-isolated (n = 613)		isolated (n = 59)		p
	N	%	n	%		n	%	n	%	
Age: Mean (SD)	73.9	(4.9)	75.6	(6.0)	0.027	74.0	(5.1)	74.7	(5.1)	0.337
Type of social participation										
Community	104	32.2%	15	20.0%	0.038	204	33.3%	11	18.6%	0.021
Senior club	43	13.3%	6	8.0%	0.207	110	17.9%	5	8.5%	0.065
Hobby	115	35.6%	15	20.0%	0.009	305	49.8%	21	35.6%	0.038
Sports	83	25.7%	7	9.3%	0.002	211	34.4%	13	22.0%	0.054
Volunteer	29	9.0%	3	4.0%	0.153	63	10.3%	1	1.7%	0.032
Politics	25	7.7%	1	1.3%	0.040	23	3.8%	1	1.7%	0.360
Industry	41	12.7%	4	5.3%	0.070	25	4.1%	1	1.7%	0.315
Religion	12	3.7%	1	1.3%	0.477	41	6.7%	1	1.7%	0.099
Number of types of organizations										
0	97	30.0%	38	50.7%	<0.001	127	20.7%	27	45.8%	<0.001
1	94	29.1%	27	36.0%		198	32.3%	15	25.4%	
2	70	21.7%	6	8.0%		148	24.1%	13	22.0%	
> = 3	62	19.2%	4	5.3%		140	22.8%	4	6.8%	
Self-reported health: good	284	87.9%	62	82.7%	0.223	532	86.8%	44	74.6%	0.010
Chronic conditions: >1 condition	267	82.7%	63	84.0%	0.782	504	82.2%	52	88.1%	0.251
Frequency of going outdoors: once a week or less	11	3.4%	4	5.3%	0.307	21	3.4%	2	3.4%	0.672
Family structure: living alone	50	15.5%	11	14.7%	0.860	173	28.2%	13	22.0%	0.310
Perceived financial status: hard	54	16.7%	14	18.7%	0.686	83	13.5%	10	16.9%	0.469
IADL disability: Mean (SD)	0.15	(0.48)	0.19	(0.46)	0.570	0.06	(0.38)	0.10	(0.40)	0.449
Emotional support: Mean (SD)	1.7	(0.9)	1.3	(0.8)	0.002	2.0	(1.0)	1.5	(0.8)	<0.001
Instrumental support: Mean (SD)	1.3	(0.7)	1.2	(0.6)	0.436	1.4	(0.8)	1.0	(0.6)	<0.001

SD: standard deviation, IADL: instrumental activities of daily living.

Table 2 presents the adjusted ORs and 95% CIs for isolation at FL among men. In the multiple logistic regression model with all covariates adjusted (Model 3), participation in a hobby groups and sports groups significantly reduced isolation among men (Table 2). Moreover, participation in two organization types and three or more organization types significantly lowered the risk of isolation, compared to non-participants (Table 2). Table 3 shows the adjusted ORs and 95% CIs for isolation at FL among women. Model 3 showed no significant association between a particular type of group and isolation among women (Table 3). However, participation in one organization type and three or more organization types significantly reduced isolation compared to non-participants (Table 3). There was a significant linear trend between the number of organization types and isolation, regardless of gender (Tables 2 and 3).

**Table 2:** Adjusted odds ratios (95% confidence intervals) for isolation at follow-up (men).

	Model 1			Model 2			Model 3		
	OR	(95% CI)	p	OR	(95% CI)	p	OR	(95% CI)	p
Type of social participation (reference: nonparticipation of each group)*									
Community	0.53	(0.29–0.97)	0.040	0.61	(0.31–1.18)	0.142	0.61	(0.31–1.22)	0.163
Senior club	0.57	(0.23–1.38)	0.212	0.70	(0.26–1.86)	0.470	0.81	(0.30–2.19)	0.678
Hobby	0.45	(0.25–0.83)	0.011	0.53	(0.28–1.01)	0.052	0.52	(0.27–0.99)	0.048
Sports	0.30	(0.13–0.67)	0.004	0.39	(0.17–0.91)	0.030	0.38	(0.16–0.91)	0.029
Volunteer	0.42	(0.13–1.43)	0.165	0.69	(0.20–2.45)	0.567	0.66	(0.18–2.42)	0.529
Industry	0.39	(0.13–1.12)	0.079	0.46	(0.15–1.35)	0.156	0.50	(0.17–1.53)	0.225
Number of types of organizations									
0	1.00			1.00			1.00		
1	0.73	(0.42–1.30)	0.285	0.72	(0.40–1.27)	0.254	0.73	(0.40–1.32)	0.292
2	0.22	(0.09–0.55)	0.001	0.21	(0.08–0.53)	0.001	0.21	(0.08–0.55)	0.001
> = 3	0.17	(0.06–0.48)	0.001	0.16	(0.05–0.47)	0.001	0.18	(0.06–0.55)	0.003
Test for linear trend							0.54	(0.40–0.72)	<0.001

*We didn't analyze the activities of only 1 participant among the isolated men.

Model 1: Crude

Model 2: Adjusted for age. Additionally, participation in all 6 organizations is added only for “type of social participation.”

Model 3: Adjusted for the covariates in Model 2 plus self-reported health, chronic conditions, IADL disability, frequency of going outdoors, family structure, perceived financial status, emotional support, and instrumental support.

OR: odds ratio, CI: confidence interval

**Table 3:** Adjusted odds ratios (95% confidence intervals) for isolation at follow-up (women).

	Model 1			Model 2			Model 3		
	OR	(95% CI)	p	OR	(95% CI)	p	OR	(95% CI)	p
Type of social participation (reference: nonparticipation of each group)*									
Community	0.46	(0.23–0.90)	0.024	0.58	(0.29–1.18)	0.134	0.68	(0.33–1.39)	0.289
Senior club	0.42	(0.17–1.08)	0.073	0.52	(0.19–1.41)	0.199	0.58	(0.21–1.58)	0.282
Hobby	0.56	(0.32–0.97)	0.040	0.67	(0.38–1.19)	0.168	0.75	(0.41–1.36)	0.338
Sports	0.54	(0.29–1.02)	0.057	0.65	(0.33–1.25)	0.192	0.78	(0.39–1.55)	0.481
Number of types of organizations									
0	1.00			1.00			1.00		
1	0.36	(0.18–0.70)	0.003	0.36	(0.19–0.71)	0.003	0.40	(0.20–0.80)	0.010
2	0.41	(0.21–0.83)	0.014	0.42	(0.21–0.85)	0.016	0.51	(0.24–1.06)	0.069
> = 3	0.13	(0.05–0.40)	<0.001	0.14	(0.05–0.40)	<0.001	0.21	(0.07–0.65)	0.006
Test for linear trend							0.64	(0.48–0.87)	0.004

*We didn't analyze the activities of only 1 participants among isolated women.

Model 1: Crude

Model 2: Adjusted for age. Additionally, participation in all 6 organizations is added only for “type of social participation.”

Model 3: Adjusted for the covariates in Model 2 plus self-reported health, chronic conditions, IADL disability, frequency of going outdoors, family structure, perceived financial status, emotional support, and instrumental support.

OR: odds ratio, CI: confidence interval

## Discussion

In developed countries, the trend toward nuclear families are progressing and the risk of isolation among older people is increased. Therefore, there are common needs to clarify possible predictors of isolation and to implement effective measures to counteract this phenomenon. The novelty of this study lies in proving the possible protective effect of social participation on future isolation from the perspective of activity types and the number of organization types. This study shows that participation in hobby or sports groups reduced the risk of isolation among older men. Furthermore, our results show that participation in two or more organizations for men and a minimum of one organization for women is effective in reducing isolation.

Concerning the types of activity, the hobby and sports groups were effective in reducing isolation among men. In addition to decreasing the risk of isolation, previous studies have reported that participation in hobby and sports groups decreases the risk of functional disability [30, 31]. Additionally, participants of hobby and sports groups have a better health-related quality of life as well as social relationships than non-participants [36, 42]. Since the current study showed that these activities are also effective in reducing isolation, we posit that encouraging older people to participate in hobby or sports groups can reduce isolation in older men. However, since the participation rate for these groups is lower in men than in women [36, 43], clarification concerning the characteristics and the reasons for non-participation is essential to determine how to encourage men to participate. Conversely, participation in community-based activities—such as neighborhood community associations and senior club—did not prove to be associated with isolation. This is consistent with Kanamori’s (2014) report concerning activity types and functional disability [30]. Previous studies have pointed out that community-based activities may be a psychological burden associated with the inevitable relationships due to the perceived bond of community [38]. Furthermore, social participation can occasionally have a negative influence on older people’ health, especially when they are forced to participate [36]. Participants in neighborhood community associations and senior club were more likely to feel as if their participation is obligatory than those who participate in hobby or sports groups [36]. This autonomy for participation may also affect the association between type of activity and isolation. There was no association between a specific type of group activity and isolation in women.

The results of the analysis of the relationship between the number of organization and isolation showed that the risk of isolation decreased with an increased number of organizations in which subjects participated for both genders. Previous studies reported that multiple group memberships lowered the risk of functional disability and contributed towards maintaining mental health [28, 30, 44]. The results of this study are in line with these findings. Lam et al. (2018) argued that participation in multiple organizations is a psychosocial resource that protects older people from threats to their health due to changes in their social identity [44]. A decline in an individual’s social role due to advancing age—which is one of main changes in social identity later in life—may lead to isolation. Therefore, multiple group memberships may contribute toward reducing isolation. Furthermore, belonging to multiple organizations increases the amount of social interaction with other individuals such as other group members. Our findings indicate that it is essential to maintain an amount of social interaction to reduce isolation.

Similar to the observations of a number of previous studies on social participation and health outcomes among older people, gender differences were also observed in this study. Some studies have showed that social participation is more effective for men than for women. According to Aida et al. (2011), participation in a volunteer group reduces the mortality risk in men but not in women [23]. Another study showed that men could gain health benefits from more varied types of social activity than women [33]. Conversely, other studies have shown that social participation is more effective for women. For example, participation in hobbies or recreational activities have been reported to support health [34], participation in volunteer activities or senior club alleviates psychological stress [32], and performing key roles in the organization reduces depression [35]. These associations, however, were found in women but not in men. It has been reported that women are more positively affected by social participation than men in terms of depression [35] and IADL [31, 45]. This study provides new findings to add to the existing literature. Since Japanese older men are more inactive than women concerning activities based on interpersonal relationships [46], the gender differences should be taken into account when encouraging regular participation in group activities.

One of the limitations of study, though, is that it covered only one small geographical area. Since the proportion of isolated older people and the subscription rate to community-based activities differ according to city size, these findings cannot be easily generalized to other areas. The novelty and value of this study, however, is in the clarification of the protective effect of social participation on isolation, which adds substantial value to the theory.

## Conclusion

Social participation lowers the risk of future isolation in Japanese older people living in an urban area. Particularly, participation in hobby groups and sports groups were effective among men. Concerning women, participation in one or more organizations is more important than the specific organization type. Encouraging participation in social activities could help reduce negative health outcomes associated with social isolation later in life.

## Supporting information

S1 File Questionnaire. (DOCX)
