# Supplementary material for: Social participation reduces isolation among Japanese older people in urban area: A 3-year longitudinal study
Source: PLoS One. 2019 Sep 20;14(9):e0222887. doi: 10.1371/journal.pone.0222887 (PMC6754169; doi:10.1371/journal.pone.0222887)
Supplement: S1 File — (DOCX) [file pone.0222887.s001.docx]

**QUESTIONAIRE**

[Social isolation]

Question 1: How often do you meet or go out with your family members who do not live together?

Question 2: How often do you talk on the phone or communicate via e-mail with your family members who do not live together?

Question 3: How often do you meet or go out with your friends?

Question 4: How often do you talk on the phone or communicate via e-mail with your friends?

Responses: 6–7 times a week (almost every day), 4–5 times a week, 2–3 times a week, about once a week, 2–3 times a month, about once a month, less than once a month, not at all

[Social participation]

Question: Are you a member of any of the following groups or organizations? Circle all applicable items. If not, circle “none”.

Responses: none, neighborhood community associations, senior citizen clubs, hobby groups, sports groups, volunteer groups, political organizations or groups, industrial or trade associations, religious organizations or groups, other

**質問項目**

【孤立】

質問1：別居のご家族や親戚と、会ったり、一緒に出かけたりすることはどのくらいありますか。

質問2：別居のご家族や親戚と、電話で話すことはどのくらいありますか。電子メールやファックスでのやりとりも含みます。

質問3：友人やご近所の方と、会ったり、一緒に出かけたりすることはどのくらいありますか。

質問4：友人やご近所の方と、電話で話すことはどのくらいありますか。電子メールやファックスでのやりとりも含みます。

回答：週に6,7回（ほぼ毎日）、週に4,5回、週に2,3回、週に1回くらい、月に2,3回、月に1回くらい、月に1回より少ない、まったくない

【社会参加】

質問：あなたは、次のようなグループや団体に入っていますか。あてはまるもの全てに○、入っていない場合は、「入っていない」に○をつけてください。

回答：入っていない、町内会・自治会、老人会・老人クラブ、趣味関係のグループ、スポーツ関係のグループやクラブ、ボランティアのグループ、政治関係の団体や会、業界団体・同業者団体、宗教関係の団体や会、その他のグループや団体
